# Ferumoxytol nanozymes effectively target chronic biofilm infections in apical periodontitis

**DOI:** 10.1172/JCI183576

**Published:** 2024-11-26

**Authors:** Alaa Babeer, Yuan Liu, Zhi Ren, Zhenting Xiang, Min Jun Oh, Nil Kanatha Pandey, Aurea Simon-Soro, Ranran Huang, Bekir Karabucak, David P. Cormode, Chider Chen, Hyun Koo

**Affiliations:** 1 Department of Endodontics, School of Dental Medicine and; 2 Biofilm Research Laboratories, Levy Center for Oral Health, School of Dental Medicine, University of Pennsylvania, Philadelphia, Pennsylvania, USA.; 3 Department of Oral Biology, King Abdulaziz University, Jeddah, Saudi Arabia.; 4 Department of Preventive and Restorative Sciences, School of Dental Medicine,; 5 Center for Innovation & Precision Dentistry, School of Dental Medicine, School of Engineering and Applied Sciences,; 6 Department of Chemical and Biomolecular Engineering, School of Engineering and Applied Sciences, and; 7 Department of Radiology, Perelman School of Medicine, University of Pennsylvania, Philadelphia, Pennsylvania, USA.; 8 Department of Stomatology, Faculty of Dentistry, University of Seville, Seville, Spain.; 9 Department of Oral and Maxillofacial Surgery and Pharmacology, School of Dental Medicine, University of Pennsylvania, Philadelphia, Pennsylvania, USA.

**Keywords:** Infectious disease, Bacterial infections, Drug therapy, Nanotechnology

## Abstract

Bacterial biofilms are pervasive and recalcitrant to current antimicrobials, causing numerous infections. Iron oxide nanozymes, including an FDA-approved formulation, ferumoxytol (FMX), show potential against biofilm infections via catalytic activation of hydrogen peroxide (H_2_O_2_). However, clinical evidence regarding the efficacy and therapeutic mechanisms of FMX is lacking. Here, we investigate whether FMX nanozymes can treat chronic biofilm infections and compare their bioactivity to that of the gold standard sodium hypochlorite (NaOCl), a potent but caustic disinfectant. Clinical performance was assessed in patients with apical periodontitis, an intractable endodontic infection affecting half of the global adult population. Data show robust antibiofilm activity by a single application of FMX with H_2_O_2_ achieving results comparable to those seen with NaOCl without adverse effects. FMX binds efficiently to the bacterial pathogens *Enterococcus faecalis* and *Fusobacterium nucleatum* and remains catalytically active without being affected by dental tissues. This allows for effective eradication of endodontic biofilms via on-site free radical generation without inducing cytotoxicity. Unexpectedly, FMX promotes growth of stem cells of the apical papilla (SCAPs), with transcriptomic analyses revealing upregulation of proliferation-associated pathways and downregulation of cell cycle suppressor genes. Notably, FMX activates SCAP pluripotency and WNT/NOTCH signaling that induces its osteogenic capacity. Together, these results show that FMX nanozymes are clinically effective against severe chronic biofilm infection with pathogen targeting and unique stem cell–stimulatory properties, offering a regenerative approach to antimicrobial therapy.

## Abstract 2

Ferumoxytol nanozymes safely eradicate severe chronic biofilm infection in apical periodontitis and promote stem cell growth, offering a new regenerative approach to antimicrobial therapy.

## Introduction

Bacterial pathogens form intractable biofilms notoriously resistant to antibiotics that cause various chronic infections and inflammation (1, 2). Apical periodontitis, a chronic and intractable dental infection affecting 52% of the adult population worldwide, is a leading cause of tooth loss and is increasingly associated with cardiovascular diseases and adverse pregnancy outcomes (3–5). Biofilms in the tooth (root) canal are the primary causative agents, and effective antibiofilm measures are crucial to mitigate these negative oral and systemic consequences (4, 6, 7). However, conventional modalities based on available antimicrobials have limited efficacy to eliminate biofilms, leading to failure of endodontic therapy (7–9). This is primarily due to inherent biofilm recalcitrance and limited drug accessibility to the biofilms formed within the anatomically complex root canal systems (10). Thus, there is an urgent need for feasible new strategies with enhanced antibiofilm efficacy.

Sodium hypochlorite (NaOCl) remains the current gold standard for endodontic disinfection owing to its strong antimicrobial and tissue dissolution properties (11). However, NaOCl is a harsh chemical that can cause severe complications if extruded to surrounding tissues and negatively affect the physical properties of dentinal tissue (12, 13). Furthermore, NaOCl is buffered by the organic tissue content within the root canal, which hampers its antimicrobial effect during treatment (14). Chlorhexidine, another potent broad-spectrum antimicrobial irrigant, is used either alone or as an adjunct to NaOCl disinfection. However, its inability to disrupt biofilm extracellular polymeric substances (EPSs) and penetrate biofilms remains a major limitation (15, 16).

Nanotechnologies have been explored to address current therapeutic limitations, with the premise that antimicrobial efficacy can be achieved given the small size and bioactivity of nanoparticles allowing access to and killing of biofilms (17). In particular, iron oxide nanoparticles have demonstrated antimicrobial activity against multiple pathogens with EPS degradation properties and biocompatibility in vivo. Moreover, US Food and Drug Administration–approved (FDA-approved) formulations have been used systemically for magnetic resonance imaging and treatment of iron deficiency anemia (18–20). Among them is ferumoxytol (FMX), an aqueous solution composed of iron oxide nanoparticles with a hydrophilic carboxymethyl-dextran coating (21). The iron oxide (Fe_3_O_4_) core of FMX exhibits a peroxidase enzyme–like property that efficiently catalyzes the reduction of hydrogen peroxide (H_2_O_2_) to generate reactive oxygen species with potential therapeutic applications in antibiofilm therapy (18). Recently, we and others have demonstrated that nanozymes can effectively target pathogenic biofilms in animal models (22–26). However, clinical evidence of antibiofilm efficacy in diseased patients is lacking.

Here, we conducted the first clinical study to investigate a nanozyme-based therapy using FMX against a severe chronic disease caused by biofilm infection. The efficacy of FMX/H_2_O_2_ was assessed in diseased patients with apical periodontitis, in whom disinfection procedures are most challenging. Data reveal near-complete disinfection by a single topical application of FMX/H_2_O_2_, achieving results comparable to those seen with the gold standard NaOCl without adverse effects. Using laboratory, ex vivo, and clinical methods, we show that FMX is a potent antibiofilm agent via on-site activation of H_2_O_2_, while unexpectedly promoting stem cell proliferation. Low doses of FMX (0.6%) and H_2_O_2_ (3%) eradicated mixed-species biofilms containing the endodontic microbes *Enterococcus* *faecalis* (a drug-resistant pathogen associated with nosocomial infections; ref. 27) and *Fusobacterium* *nucleatum* (linked with several forms of cancer; ref. 28). FMX nanozymes can bind to biofilm and its bacterial constituents and display peroxidase-like activity in situ within the root canal. Notably, FMX was devoid of cytotoxicity or caused apoptosis but induced several growth-stimulatory genes and proliferation and osteogenic differentiation of apical papilla stem cells. Our data demonstrate a readily translatable therapeutic platform to combat biofilm-associated infections with tissue regeneration potential.

## Results

### Assessment of clinical treatment regimen.

**Figure 1:**
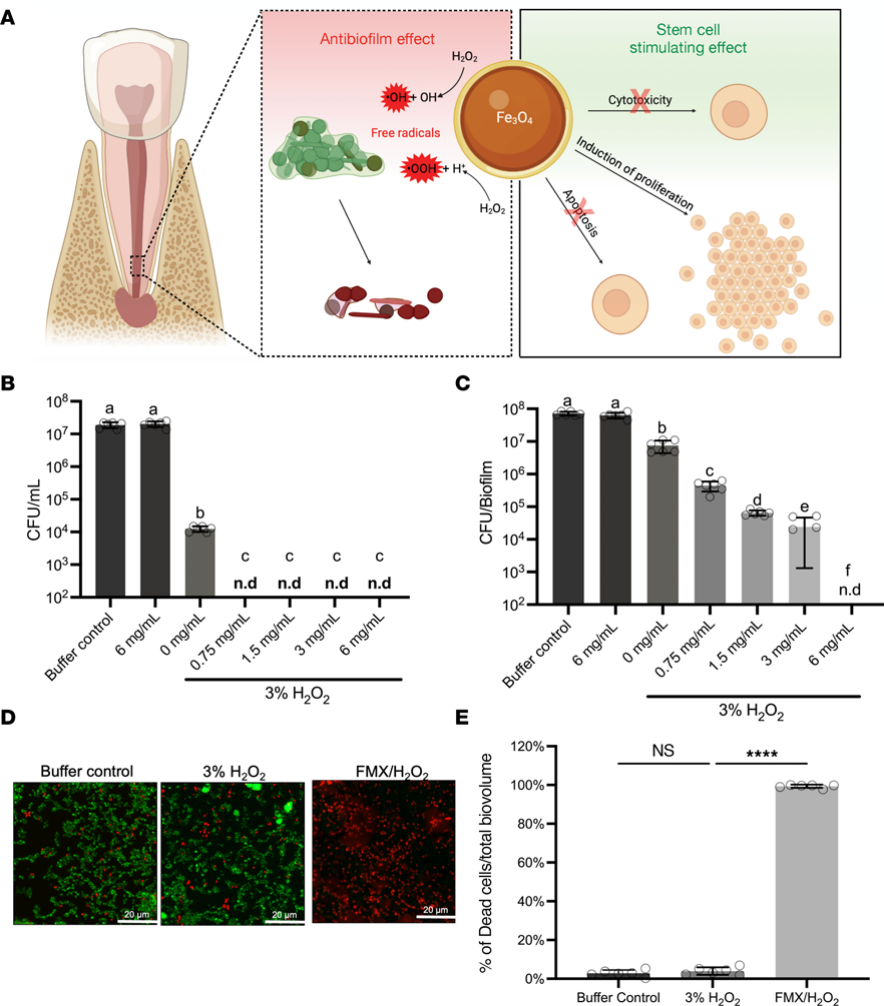
FMX nanozymes eliminate drug-resistant bacterial biofilms associated with severe endodontic infection through catalytic activation of H_2_O_2_. (**A**) A schematic depiction of antibiofilm activity and stem cell–stimulatory effects of FMX (Created in BioRender. Babeer, A. (2025) https://BioRender.com/y71u571). (**B**) Dose-dependent antimicrobial activity of FMX/H_2_O_2_ against planktonic *E*. *faecalis* cells after 10 minutes (n = 6). (**C**) Dose-dependent efficacy of FMX/H_2_O_2_ against *E*. *faecalis* biofilm after 10 minutes (n = 6). Different letters indicate statistically significant differences. (**D**) Representative confocal images of *E*. *faecalis* biofilm. Live cells were stained with SYTO 9 (green), and dead cells were stained with propidium iodide (red). Scale bars: 20 μm. (**E**) Relative percentage of live and dead cells of the total biovolume (n = 7). The statistical analysis was performed using ANOVA, followed by Tukey’s test for multiple comparisons. All values are reported as mean ± SD, ****P < 0.0001; ns, not significant; n.d., non-detectable.

To establish the groundwork for a clinical application of the nanozyme-mediated treatment (Figure 1A), we conducted a series of experiments under conditions simulating the clinical environment. We first assessed whether the catalytic activity of FMX was affected by the presence of dental tissues inside the root canal. The iron oxide (Fe_3_O_4_) core of FMX nanozymes can catalyze H_2_O_2_ to release free radicals in a peroxidase-mimic fashion, which can be assayed using colorimetric substrates, such as 3,3′,5,5′-tetramethylbenzidine (TMB) (22). The results show that FMX rapidly catalyzed the release of bioactive free radicals in a dose-dependent manner (Supplemental Figure 1A; supplemental material available online with this article; https://doi.org/10.1172/JCI183576DS1) (*P* < 0.001). The presence of dental tissues had negligible effect on its catalytic activity (Supplemental Figure 1B) (*P* > 0.05), demonstrating the feasibility of using FMX in the endodontic microenvironment.

Next, we sought to identify the optimal FMX concentration and treatment time against *E*. *faecalis*, a pathogen commonly isolated from persistent apical periodontitis and known for its antimicrobial resistance (27, 29). To determine the dose- and time-dependent antimicrobial effects, we exposed actively growing *E*. *faecalis* to different concentrations of FMX in conjunction with 3% H_2_O_2_, a clinically used dosage. Data showed that all tested FMX concentrations (0.75 to 6 mg/mL) effectively eradicated *E*. *faecalis* when combined with 3% H_2_O_2_ after a 10-minute treatment, outperforming 3% H_2_O_2_ alone (*P* < 0.0001). In contrast, the H_2_O_2_ resulted in only a moderate reduction of bacterial viability compared with the control (*P* < 0.0001) and failed to fully eradicate the pathogen (Figure 1B). We also evaluated the bioactivity of FMX/H_2_O_2_ with shorter exposure times. While 1-minute treatments were unable to kill bacteria (*P* > 0.05), a 5-minute treatment with 6 mg/mL FMX successfully eradicated *E*. *faecalis* (*P* < 0.0001 vs. H_2_O_2_ alone) (Supplemental Figure 2, A and B).

Given that microbes within biofilms exhibit heightened antimicrobial tolerance (2, 30), we further tested FMX’s potency against mature *E*. *faecalis* biofilms using an established HA-coated peg model (MBEC Assay) (31). Biofilms were treated with a single FMX/H_2_O_2_ topical application, simulating a treatment protocol feasible in a clinical setting. *E*. *faecalis* biofilms showed high tolerance to H_2_O_2_ alone, with less than 1-log reduction in viable cells after 10 minutes. In contrast, we found a marked reduction in viable counts with increasing amounts of FMX combined with H_2_O_2_ (*P* < 0.0001), culminating at 6 mg/mL FMX, whereby complete biofilm eradication was achieved with no detectable viable cells (Figure 1C), demonstrating the crucial role of FMX in catalytically enhancing H_2_O_2_’s antimicrobial effect. As expected, shorter treatment durations (1 and 5 minutes) were less effective against *E*. *faecalis* biofilms (Supplemental Figure 3, A and B). We also assessed the cell viability within intact biofilms using in situ confocal microscopy combined with live/dead cell labeling and quantitative computational analysis (32). Data showed widespread killing of *E*. *faecalis* cells across the biofilm structure by FMX/H_2_O_2_, whereas bacteria were only sparsely killed by H_2_O_2_ alone (Figure 1, D and E). These results indicate that FMX nanozymes can eliminate drug-resistant bacteria associated with severe endodontic infection residing within biofilms through catalytic activation of H_2_O_2_.

### Bioactivity of FMX against ex vivo mixed-species biofilm.

**Figure 2:**
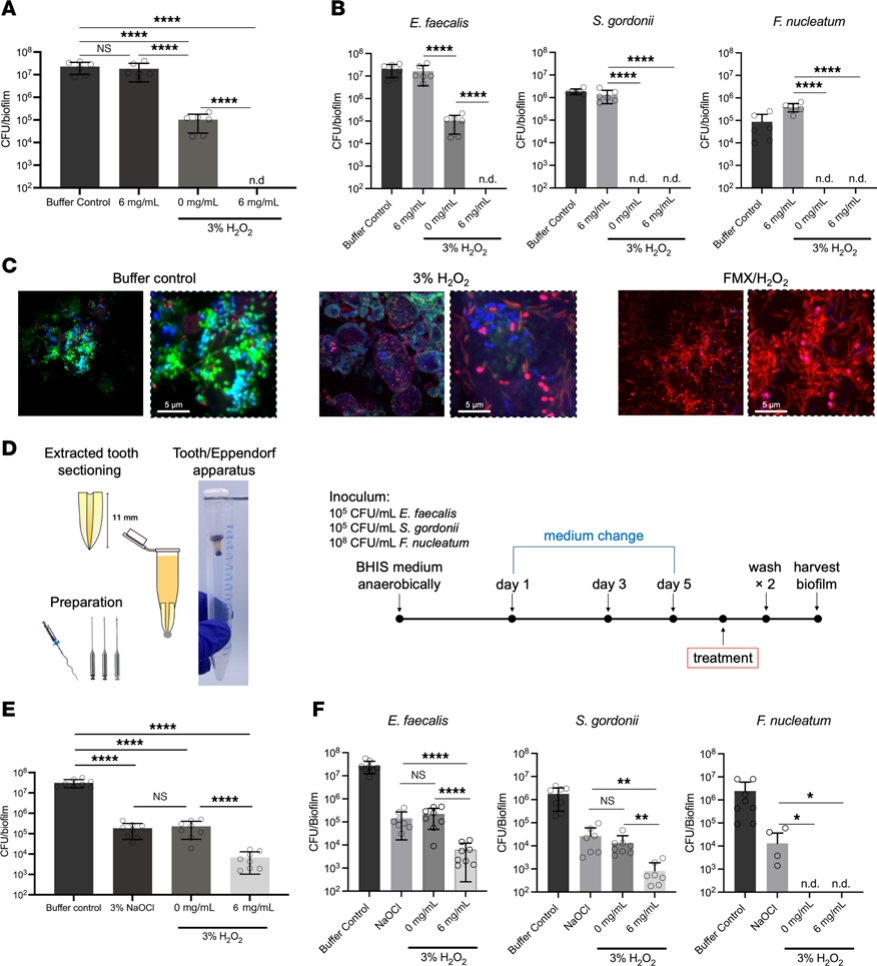
Bioactivity of FMX nanozymes on in vitro and ex vivo mixed-species biofilms. (**A**) Antibiofilm effect of FMX/H_2_O_2_ on the total viable bacteria in the in vitro biofilm model (*n* = 6). (**B**) The effect of FMX/H_2_O_2_ on the viability of different bacterial species in in vitro mixed-species biofilms (*n* = 6). (**C**) Representative images of mixed-species biofilms. All samples were stained with SYTO 60 (green) or propidium iodide (red); GFP-labeled *E*. *faecalis* cells are shown in blue. Dashed squares highlight zoomed-in areas within the micrographs. Scale bars: 5 μm. (**D**) A schematic of the ex vivo biofilm model using extracted human tooth. BHIS, Brain heart infusion-supplemented. (**E**) Antibiofilm effect of FMX/H_2_O_2_ on the total number of viable bacteria in the mixed-species biofilm (*n* = 6). (**F**) The effect of FMX/H_2_O_2_ on the viability of different species in ex vivo mixed-species biofilms (*n* = 6). The statistical analysis was performed using ANOVA followed by Tukey’s test for multiple comparisons (**A**, **B**, **E**, and **F**, left panel) or Kruskal-Wallis test followed by Dunn’s test for multiple comparisons (**F**, middle and right panels). All quantitative values are reported as mean ± SD, *P < 0.05, **P < 0.01, ****P < 0.0001; ns, not significant; n.d., non-detectable.

Endodontic infections are characterized by different species coexisting within a structurally complex root canal system (9, 10, 33). To further assess FMX efficacy, we used a mixed-species biofilm model incorporating *E*. *faecalis*, *Streptococcus gordonii*, and *F*. *nucleatum*, which are frequently isolated from persistent endodontic infections and are linked to various systemic conditions (27, 28, 33). The results show that a 10-minute treatment of FMX (6 mg/mL) with H_2_O_2_ effectively eradicated the biofilm with no detectable viable cells, outperforming H_2_O_2_ alone (*P* < 0.0001) (Figure 2A). Further taxonomy analysis revealed that while H_2_O_2_ was able to eradicate *S*. *gordonii* and *F*. *nucleatum*, it failed to kill *E*. *faecalis*. Conversely, FMX with H_2_O_2_ effectively eliminated *E*. *faecalis* together with other species from the mixed biofilm (*P* < 0.0001) (Figure 2B). This result was further confirmed by confocal microscopy, demonstrating antimicrobial action against all species within the biofilm structure (Figure 2C).

To gain insights into FMX performance in a clinically simulated setting, we utilized an ex vivo biofilm model using extracted human teeth (34). This model allows direct comparison of FMX/H_2_O_2_ efficacy against the gold standard 3% NaOCl solution under conditions that closely resemble clinical antimicrobial irrigation in endodontic treatment (Figure 2D). The nanozyme-based treatment was significantly more effective (>100-fold) than either NaOCl or H_2_O_2_ alone (*P* < 0.0001) (Figure 2E). Intriguingly, FMX achieved significantly higher reduction of *E*. *faecalis* (*P* < 0.0001) and *S*. *gordonii* (*P* < 0.01) compared with NaOCl or H_2_O_2_, while eradicating *F*. *nucleatum* (Figure 2F), demonstrating potent biocidal activity against mixed-species biofilm grown in the root canal.

### Cytotoxicity of FMX on stem cells of the apical papilla.

**Figure 3:**
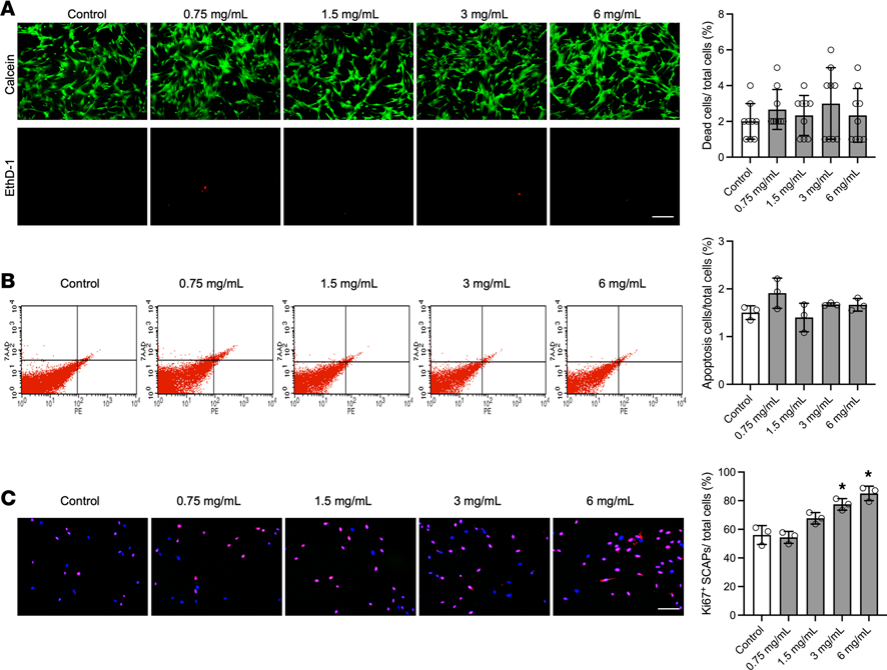
FMX at antimicrobial dosages promotes cell proliferative capabilities without cytotoxicity in human stem cells of the apical papilla. (**A**) Cell viability assay showed that topical exposure of FMX did not induce cell death in stem cells of the apical papilla (SCAPs). Scale bar: 25 μm. (**B**) Flow cytometry analysis showed that FMX did not induce cell apoptosis in SCAPs. (**C**) Cell proliferation assay showed that FMX treatment significantly increased Ki67^+^ cell percentage in SCAPs. Scale bar: 25 μm. Quantitative data are shown at the right of each panel. The statistical analysis was performed using ANOVA, followed by Dunnett’s test for multiple comparisons. All quantitative values are reported as mean ± SD, *P < 0.05.

Demonstrating antimicrobial potency while ensuring biocompatibility with the adjacent host cells represents another essential yet unattainable feature of current disinfecting agents (35, 36). We next examined FMX’s impact on the viability and proliferative potential of stem cells of the apical papilla (SCAPs), a population of mesenchymal stem-like cells residing in direct contact with the tooth apex and critical for periapical healing (37). Human apical papilla tissues were obtained from third molars with immature roots of healthy patients (aged 16–20 years) as discarded biological samples from the dental clinic of the University of Pennsylvania. Post-treatment viability assays of SCAPs exposed to varying FMX concentrations revealed no cytotoxic effects (*P* > 0.05) (Figure 3A). Moreover, flow cytometry analyses confirmed that FMX did not induce early- or late-stage apoptosis markers (annexin V–positive/7-aminoactinomycin D [7-AAD]–negative and annexin V–positive/7-AAD–positive) in SCAPs (*P* > 0.05) (Figure 3B). However, we found that FMX at concentrations of 3 and 6 mg/mL significantly increased SCAP proliferation, as determined by Ki67 immunofluorescence staining (Figure 3C). Collectively, these findings guided us for the selection of 6 mg/mL FMX and 10-minute treatment duration for the clinical study. This regimen was selected to maximize antibiofilm effectiveness while minimizing any potential cytotoxic effects.

### Clinical assessment of FMX nanozyme catalysis-based approach.

**Figure 4:**
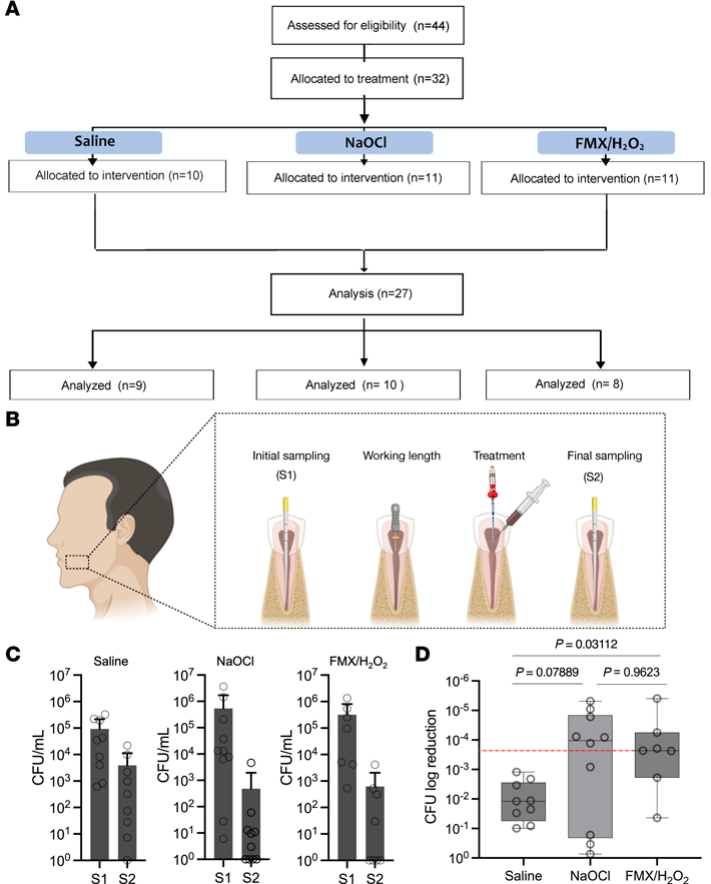
Clinical effectiveness of FMX/H_2_O_2_. (**A**) A flowchart of the clinical study. (**B**) A simplified schematic diagram of the clinical treatment regimen and sample collection. Created in BioRender. Babeer, A. (2025) https://BioRender.com/o72b703 (**C**) The number of colony-forming units (CFU) before and after treatments. Values are reported as mean ± SD. (**D**) Antimicrobial effectiveness of different clinical protocols. Values are reported in box plots and were subjected to Mann-Whitney *U* test for pairwise comparisons. *P* values are represented within the graph. Red dashed line highlights the relationship of the median of log reduction of FMX/H_2_O_2_ relative to the median log reduction of NaOCl.

Guided by antibiofilm performance data, we formulated an irrigation solution containing FMX nanozymes and H_2_O_2_ for a single treatment of root canal infections in patients with apical periodontitis, a condition characterized by chronic inflammation caused by endodontic biofilm infection. An overview of the clinical study design is presented in Figure 4A. A detailed study flowchart, inclusion/exclusion criteria, clinical protocols, treatment allocation, and randomization processes are provided in Supplemental Methods and Supplemental Figure 4.

Patients were evenly randomized into 3 groups: FMX/H_2_O_2_ (test group), saline (negative control), and NaOCl (positive control), as illustrated in the treatment protocol schematic (Figure 4B). We opted to exclude H_2_O_2_ alone, since the results of benchtop studies showed limited efficacy (Figure 1C and Figure 2, A and E), consistent with low antibiofilm activity of H_2_O_2_ against biofilms shown in previous studies (18, 25). The teeth were subjected to standard endodontic surgical procedures prior to the FMX/H_2_O_2_ treatment, including field isolation, caries removal, and mechanical preparation of the root canal. An initial (pretreatment) sample (S1) was taken using a sterile paper point inserted into the canal and then stored in liquid dental transport medium optimized to preserve microbial viability. For clinical use, FMX and H_2_O_2_ were pre-mixed using 2 syringes attached in tandem to create ready-to-use treatment solution chair-side immediately before application (Supplemental Video 1). The treatment solution (saline, NaOCl, or FMX/H_2_O_2_) was then administered within the root canal during endodontic instrumentation and kept for 10 minutes followed by solution removal. A second (post-treatment) sample (S2) was collected using sterile paper points. S1 and S2 samples were immediately processed to determine bacterial viability.

The viable bacterial counts recovered from S1 and S2 for each group are shown in Figure 4C. The FMX/H_2_O_2_ group demonstrated remarkable antimicrobial efficacy, achieving 99.9% reduction in bacterial counts (>3 logs reduction), consistent with the data from the ex vivo biofilm model (Figure 2E). As expected, saline (negative control) achieved only limited reduction in viable bacterial counts (Figure 4C), consistent with previous findings (38). Importantly, the antimicrobial effectiveness of FMX/H_2_O_2_, measured as total CFU log reduction, was comparable to that of NaOCl, the clinical gold standard treatment (Figure 4D). Furthermore, no adverse effects were observed following the treatment with FMX/H_2_O_2_. Altogether, our data show the clinical effectiveness of FMX nanozyme catalysis in treating severe biofilm infection in patients with chronic apical periodontitis, demonstrating its efficacy as a safer and effective alternative to NaOCl.

### Surface binding and in situ catalytic action of FMX nanozymes.

**Figure 5:**
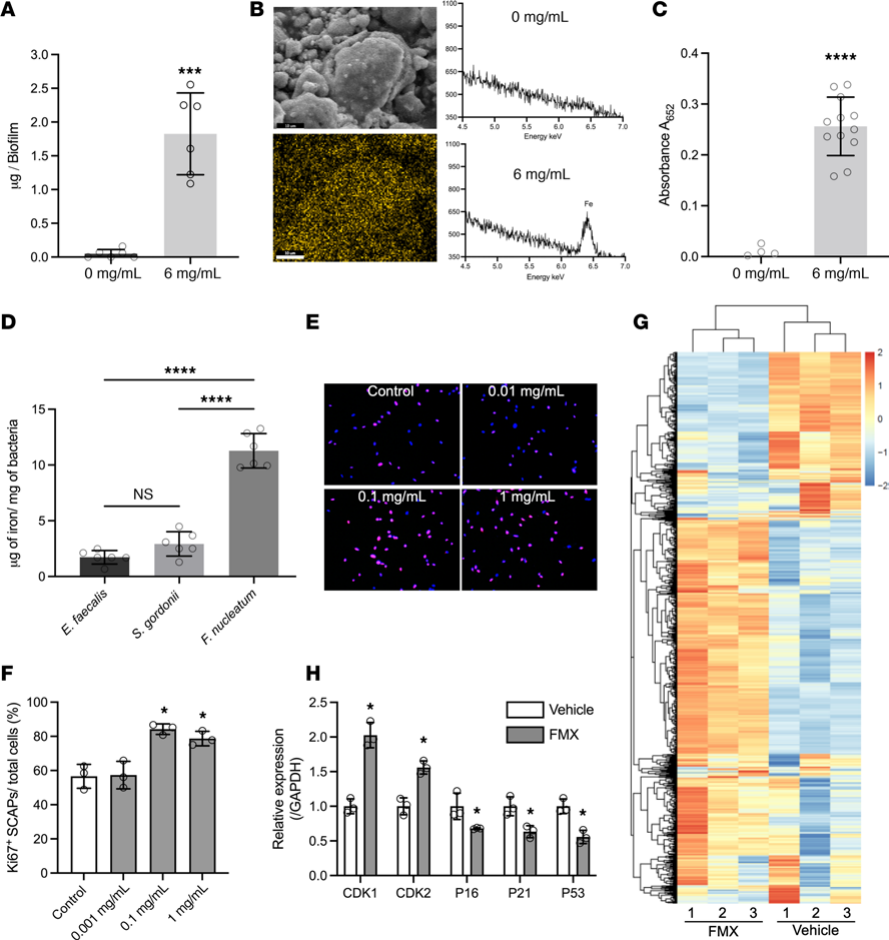
Surface retention, in situ catalysis, and regenerative potential of FMX nanozymes. (**A**) Amount of FMX retained in treated biofilms after 10 minutes of treatment measured by ICP-OES (*n* = 6). (**B**) Left: A representative environmental scanning electron microscope image of the FMX-treated biofilm and the corresponding elemental mapping image showing iron ion (yellow) distribution. Scale bars: 10 μm. Right: EDS spectra of untreated and FMX-treated biofilms. (**C**) Catalytic activity of retained FMX in biofilms (*n* = 12). (**D**) Amount of FMX bound to bacterial cells after 10 minutes of treatment (*n* = 6). (**E**) Ki67 staining showed that 0.1 mg/mL or higher concentrations of FMX significantly increased SCAP proliferation after a 24-hour treatment. Scale bar: 25 μm. Original magnification × 20. (**F**) Quantitative analysis of the percentage of Ki67^+^ cells after treatment (*n* = 3). (**G**) Heatmap of transcriptomics analysis showing genes differentially regulated after 1 mg/mL FMX treatment for 24 hours. Gene expression is shown in normalized log_2_ counts per million. Differentially expressed genes were selected based on a 4-fold change. (**H**) qPCR assay confirmed that cell cycle genes were highly activated while cell cycle suppressor genes were greatly diminished after FMX treatment. Statistical analyses were performed using Welch’s 2-tailed *t* test (**A** and **C**), ANOVA followed by Tukey’s test (**D**), or ANOVA followed by Dunnett’s test (**F** and **H**) for multiple comparisons. All values are reported as mean ± SD, *P < 0.05, ***P < 0.001, ****P < 0.0001; ns, not significant.

To further understand the clinical performance observed in the FMX trial, we conducted additional studies to provide mechanistic insights into its mode of action. Given the short lifespan and the short travel distance of free radicals (39, 40), the release of these bioactives requires retention and proximity of the nanozymes to the biofilm cell surface. We analyzed the retention and catalytic activity of surface-bound FMX within *E*. *faecalis* biofilm following the same clinical treatment protocol (6 mg/mL FMX and 10-minute treatment). Inductively coupled plasma–optical emission spectrometry (ICP-OES) was used for the quantification of FMX bound to the biofilm. The data revealed significant accumulation of nanozymes in the treated biofilms compared with the untreated control (*P* < 0.001), indicating FMX retention (Figure 5A). We further confirmed FMX binding using scanning electron microscopy coupled with energy dispersive spectroscopy (EDS). The EDS elemental mapping demonstrated iron distribution across the biofilm surface (Figure 5B, left panel), with the corresponding EDS spectra indicating iron peaks in the FMX-treated biofilms, confirming in situ retention (Figure 5B, right panel).

Next, we examined whether the retained FMX remains catalytically active after binding. After FMX treatment, biofilms were exposed to a reaction buffer (containing TMB and 3% H_2_O_2_). The results showed that surface-bound nanozymes were catalytically active, marked by the on-site generation of free radicals as determined via TMB assay (Figure 5C), which can be directly visualized as shown in Supplemental Figure 5. Collectively, the data indicate that FMX nanozymes are retained within biofilm and maintain its catalytic functions upon binding. This feature allows localized catalysis of H_2_O_2_ for a targeted and effective antimicrobial action at the site of infection.

Furthermore, we assessed the binding affinity of FMX to each bacterial species (Figure 5D). Our results show that FMX binds all 3 species with notably higher affinity to *F*. *nucleatum* over the other species (*P* < 0.0001), demonstrating a preferential binding-killing mechanism, supporting ex vivo mixed-biofilm data that show higher killing efficacy of *F*. *nucleatum* (Figure 2F). These findings suggest a potent, targeted antimicrobial action against a pathogen with systemic implications residing in complex communities in endodontic infections.

### FMX-induced SCAP proliferation and regenerative potential.

The intriguing in vitro stem cell proliferative activity with lack of cytotoxicity and adverse effects in the trial motivated us to investigate the molecular mechanisms by which FMX influences SCAPs, a key mesenchymal stem cell type in regenerative endodontics. We investigated whether long-term (24-hour) exposure to FMX could modulate the proliferation capacity of SCAPs. Interestingly, even at low concentrations (0.1 mg/mL), FMX significantly increased the percentage of Ki67^+^ SCAPs, indicating a strong cell proliferation stimulus (representative images in Figure 5E; quantification in Figure 5F). This discovery led us to perform transcriptomics analyses to identify the reasons behind FMX’s ability to boost SCAP proliferation. RNA-Seq revealed that FMX treatment significantly altered the expression of approximately 3,000 transcripts (Figure 5G). Gene set enrichment analysis further showed that gene sets associated with cell proliferation, such as microtubule cytoskeleton organization and centromere complex assembly, were notably enriched after FMX treatment (Supplemental Figure 6). Complementary quantitative PCR (qPCR) analysis supported these findings, showing a significant upregulation of cell cycle–promoting genes such as *CDK1* and *CDK2*, whereas cell cycle suppressor genes such as *p16*, *p21*, and *p53* were downregulated (Figure 5H).

### FMX induces SCAP lineage differentiation and osteogenesis.

**Figure 6:**
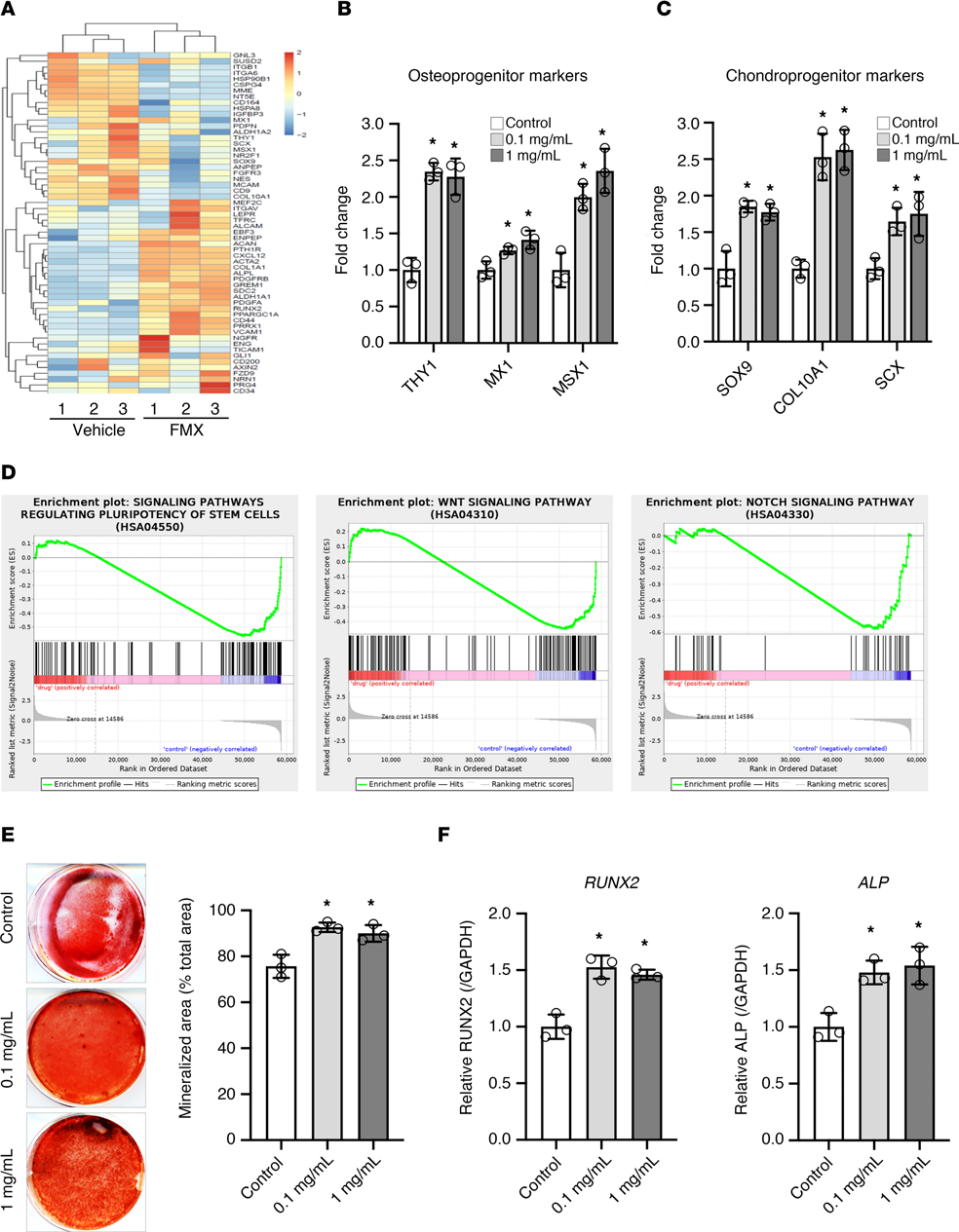
FMX promotes osteogenic differentiation in SCAPs. (**A**) Heatmap from RNA-Seq showing differential regulation of stemness genes in SCAPs following treatment with FMX. (**B** and **C**) qPCR assays demonstrating significant increases in the levels of osteogenic and chondrogenic markers in FMX-treated SCAPs (*n* = 3). (**D**) Gene set enrichment analysis indicates that osteogenic pathways, including WNT and NOTCH signaling, are enriched in FMX-treated SCAPs. (**E**) Alizarin red staining reveals enhanced osteogenic capacity of SCAPs with FMX treatment, indicating increased mineralized nodule formation (*n* = 3). (**F**) Further qPCR analysis confirms significant upregulation of the osteogenic markers RUNX2 and ALP after FMX treatment (*n* = 3). Statistical analyses were performed using ANOVA followed by Dunnett’s test for multiple comparisons (vs. the control group). All values are presented as mean ± SD, *P < 0.05.

In addition to cell proliferation, we then asked whether FMX treatment is able to affect lineage differentiation, another key feature of SCAPs. Our transcriptomic analysis showed that a low dosage (0.1 mg/mL) and long-term (24-hour) exposure to FMX largely altered the expression of mesenchymal differentiation marker genes (Figure 6A). Specifically, the levels of osteogenic markers such as *THY1*, *MX1*, and Msh homeobox-1 (*MSX1*) as well as chondrogenic markers such as SRY-box transcription factor-9 (*SOX9*), collagen type X alpha 1 chain (*COL10A1*), and scleraxis basic helix-loop-helix transcription factor (*SCX*) were significantly higher in FMX-treated SCAPs compared with the control group, as demonstrated by qPCR analysis (Figure 6, B and C). These findings were further confirmed by gene set enrichment analysis, showing enrichment of osteogenesis-related pathways including stem cell pluripotency, WNT signaling, and NOTCH signaling in FMX-treated SCAPs (Figure 6D). Motivated by these results, we assessed the osteogenic capacity of SCAPs with and without FMX treatment. Under osteogenic induction conditions, alizarin red staining showed that 0.1 to 1 mg/mL FMX treatment significantly enhanced the formation of mineralized nodules in SCAPs (Figure 6E). Furthermore, qPCR confirmed an increase in the expression levels of osteogenic genes including runt-related transcription factor-2 (*RUNX2*) and alkaline phosphatase (*ALP*) after FMX treatment in SCAPs (Figure 6F).

In sum, our findings not only affirm FMX nanozyme’s safety and efficacy in a clinical setting but also highlight its microbial binding property and ability to augment stem cell proliferation. This synergy lays the groundwork for its potential applicability in regenerative antimicrobial therapy — a dual-targeting strategy whereby biofilm disruption is coupled with the promotion of tissue regeneration.

## Discussion

Our data provide direct clinical evidence that FMX nanozymes display potent antibiofilm action with an unexpected stimulatory activity on SCAP growth and differentiation. Notably, the nanozyme-based approach is as effective as the gold standard NaOCl, a powerful bactericidal agent but with the inherent limitation of being caustic and cytotoxic. We found that FMX can bind biofilm microbes and display peroxidase-like catalysis to generate antimicrobial free radicals in situ. These features provide an effective yet targeted action against important pathogens such as *E*. *faecalis* and *F*. *nucleatum*. Moreover, FMX exhibited no cytotoxicity while promoting the expression of genes related to cell proliferation and cell cycle regulation in SCAPs and inducing osteogenic cell differentiation, marking a unique property. This dual functionality can be leveraged for regenerative antibiofilm treatment, where stem cell proliferation and functional modulation are desired after microbial disinfection to accelerate tissue regeneration (41, 42). Importantly, a single treatment using low concentrations of FMX (>50 times less than the FDA-approved systemic dosage for anemia) and H_2_O_2_ amounts typically found in over-the-counter products (43) can achieve effective disinfection in patients with severe endodontic infection. Thus, and in contrast to current treatment standard, FMX nanozymes exhibit unique antimicrobial with stem cell–stimulatory properties that could have immediate clinical applications to combat apical periodontitis with implications for treating other biofilm-related diseases.

The findings that FMX showed high efficacy in eliminating *E*. *faecalis* and *F*. *nucleatum* within biofilms are particularly noteworthy. These species, in addition to their roles in endodontic infection, have been associated with other major health care problems. *E*. *faecalis* is a common pathogen associated with human diseases including endocarditis and urinary tract and intra-abdominal infections and notoriously resistant to antimicrobials (27) including H_2_O_2_ and NaOCl as demonstrated here. The high killing efficacy of *F*. *nucleatum* may have ectopic implications due to increased association of *F*. *nucleatum* of oral origin with several forms of cancer, including head and neck, breast, and colorectal cancer (28). Two important mechanisms play integral roles in the FMX nanozyme–mediated antimicrobial activity. The effective adhesion of FMX to bacterial and biofilm surfaces, and its active catalysis without being affected by surface binding or presence of dental tissues, allow localized activation of H_2_O_2_. This combined feature is important because free radicals have limited travel distance at microscale; therefore proximity to the targeted microbes is desirable to achieve high killing efficacy (39, 40). We observed a particularly effective elimination of *F*. *nucleatum* by FMX, which can be explained in part by its higher binding affinity in comparison with other species, facilitating free radical production directly onto the bacterial surface. FMX binding may be mediated by cell surface–expressed carbohydrate binding domains in combination with electrostatic interactions (44), but further investigations are needed to elucidate specificity to *F*. *nucleatum*. These properties combined with lack of cytotoxicity (25) could facilitate the development of nanozyme-based approaches tailored to target these pathogens.

FMX promotes both growth and functional activation of SCAPs by inducing stem cell differentiation with osteogenic capacity, critical for apical healing and repair. The exact cell stimulatory mechanisms of FMX remain to be elucidated, although at least 3 different factors may be involved: (a) activation of cell proliferation and cell cycle regulation pathways, (b) activation of stem cell pluripotency and osteogenic pathways, and (c) induction of WNT and NOTCH signaling. In addition, endocytosed iron oxide nanoparticles can modulate intracellular H_2_O_2_ levels and induce cell proliferation (45). Conversely, surface coatings (e.g., dextran) on iron oxide nanoparticles could prevent cytotoxic effects and stimulate cell growth (46), although incubation of stem cells with similar dextrans did not induce proliferation (47), suggesting specific cell-surface interactions. Given that NaOCl and Chlorhexidine (CHX) are cytotoxic and can kill SCAPs (35, 36), the host cell–stimulatory effect of FMX may provide a substantial advantage for regenerative endodontic procedures in which the survival of stem cells is critical for tissue regeneration (42). Current antimicrobial agents (NaOCl and CHX) are cytotoxic to SCAPs and have deleterious effects on their survival. Hence, the use of supplemental irrigants, deactivating agents, and lower concentrations has been recommended to mitigate their harmful effects (35, 36). Given the potent antimicrobial effects of FMX-mediated catalysis of H_2_O_2_, the gentle effect on SCAPs upon transient exposure, and the proliferation-inducing effect of FMX upon prolonged exposure at low doses, there is an untapped opportunity for the development of a streamlined multifunctional therapeutic approach for regenerative endodontic procedures (48).

Despite promising results, there are limitations to our clinical study, but also opportunities for further research. The sample size and endpoint results provide limited information about the long-term effects on disease resolution and tissue repair. Given our demonstration of clinical antibiofilm efficacy, longitudinal studies with larger sample size could elucidate the bioactivity of the FMX nanozyme therapy on both periapical healing and tissue regeneration. The stem cell proliferation properties warrant further investigation. RNA-Seq data point toward cell proliferation but also stem cell differentiation, which was further supported experimentally; this may reveal additional molecular mechanisms of action. Conversely, detailed structural studies of FMX-microbe binding interactions may lead to discovery of species-specific binding domains. An important translation feature is the low-cost synthesis of iron oxide nanoparticles using simple chemistry that can be readily scalable in good manufacturing practice (GMP) facilities, which could facilitate product development for future clinical trials. Altogether, our data show a multimodal therapeutic alternative against a severe and prevalent chronic disease that could be leveraged clinically to combat biofilm infections with unique cell regeneration properties.

## Methods

### Sex as a biological variable.

Our clinical study included both male and female participants. Sex was not considered as a biological variable in the study.

### Optimization of FMX for bacterial killing and biofilm disruption.

*Enterococcus faecalis* (OG1RF), a clinical isolate from persistent apical periodontitis (a gift from Brenda Gomes, Faculdade de Odontologia de Piracicaba/UNICAMP, Piracicaba, Sao Paulo, Brazil), were grown in brain-heart infusion medium to mid-exponential phase. For antimicrobial assays, the bacterial suspension (10^7^ CFU/mL) was treated with FMX (0.75, 1.5, 3, and 6 mg/mL) in 0.1 M sodium acetate (NaOAc) buffer (pH 4.5) and 3% H_2_O_2_ at 37°C for 1, 5, and 10 minutes. Controls included 3% H_2_O_2_, FMX (6 mg/mL), or NaOAc buffer alone. Viability was assessed by counting of colony-forming units (CFU) after treatment. *E*. *faecalis* biofilms formed on hydroxyapatite-coated pegs (MBEC Assay; Innovotech Inc.) were topically treated with FMX (0.75–6 mg/mL) in 0.1 M NaOAc (pH 4.5) and 3% H_2_O_2_ for 1, 5, and 10 minutes. CFU counting and in situ confocal microscopy with LIVE/DEAD assay were performed to assess biofilm viability after treatment. Experimental details are provided in Supplemental Methods.

### In vitro and ex vivo mixed-species biofilm treatment and analysis.

Mixed-species biofilms composed of *E*. *faecalis*, *S*. *gordonii*, and *F*. *nucleatum* on hydroxyapatite-coated pegs were treated as described above, followed by species-specific CFU counting. The differentiation was done based on colony morphology. Differentiation between *E*. *faecalis* and *S*. *gordonii* was achieved with observation of α-hemolysis rings around *S*. *gordonii* colonies. *F*. *nucleatum* colonies were yellowish in color with shiny striations on the colony surface. These observations were previously confirmed with colony PCR, LIVE/DEAD assays, and confocal imaging as detailed in Supplemental Methods. An established ex vivo mixed-species biofilm model using extracted human teeth (33) was used to further assess treatment efficacy. Biofilms were treated with 0.89% NaCl, 3% NaOCl, or FMX/H_2_O_2_ for 10 minutes to evaluate FMX performance in a setting simulating a clinical setting, whereby antimicrobial agents are introduced into the canals of extracted teeth and in a manner similar to clinical application of antimicrobial irrigants. Detailed information on the methods and procedures is provided in Supplemental Methods.

### Viability and apoptosis assays of human apical papilla stem cells.

Human SCAPs were exposed to FMX for 30 minutes (short term) or 24 hours (long term) for assessing cell viability and apoptosis using a LIVE/DEAD kit (L3324, Invitrogen) and annexin V staining. Ki67 staining (559763, BD Biosciences) was performed to quantify cell proliferation after treatment. Experimental details are provided in Supplemental Methods.

### Human subject recruitment, clinical procedure, and sample collection.

Briefly, 44 patients diagnosed with periapical periodontitis were enrolled and assessed for eligibility, and then evenly randomized into 3 groups (FMX/H_2_O_2_, saline, and NaOCl). The primary endpoint of this study was the reduction in viable bacterial counts from pretreatment to post-treatment (S1 to S2) in each group, measured by colony-forming units (CFU). Standard endodontic surgery was performed prior to treatment application. An initial microbial sample (S1) was collected from the root canal using 2 sterile paper points. Treatment solutions were administered within the root canal during endodontic instrumentation and kept for 10 minutes, followed by a second (post-treatment) sample (S2) collection. Both S1 and S2 were immediately transferred to a microbiological laboratory to determine viable bacterial counts. The secondary endpoint involved comparing the magnitude of bacterial reduction between the 3 treatment groups (FMX/H_2_O_2_, saline, and NaOCl), using the Mann-Whitney *U* test for pairwise comparisons. Detailed information about inclusion/exclusion criteria, investigational new drug exemption, randomization, allocation to treatment, clinical protocol, and treatment/sampling is provided in Supplemental Methods.

### FMX nanozyme binding and catalytic activity.

The amount of FMX binding to biofilms was determined using inductively coupled plasma–optical emission spectrometry (ICP-OES) as described previously (49). Elemental analysis was performed by environmental scanning electron microscope coupled with energy dispersive spectroscopy (ESEM/EDS) to assess FMX distribution on the treated biofilm surface. Catalytic activity of bound FMX was assessed using tetramethylbenzidine-based colorimetric assay. Experimental and analytical details are described in Supplemental Methods.

### Transcriptomic analysis of human SCAPs.

RNA was isolated and prepared from the cultured human SCAPs (with or without FMX treatment) using established protocols for library preparation, fragmentation, cDNA synthesis, second-strand synthesis, PCR enrichment, and sequencing by Illumina sequencers. Reads obtained from RNA-Seq were aligned to the human reference genome to analyze differential gene expression. The differential expression between conditions was statistically assessed, and genes with fold change > 1 and fold change < 1 and *P* < 0.01 were identified as differentially expressed. Gene set enrichment analysis (GSEA) was performed using GSEA software with differentially expressed genes, followed by qPCR for data validation as described in detail in Supplemental Methods.

### Osteogenic differentiation analysis.

For in vitro osteogenic differentiation assay, SCAPs were cultured under osteogenic-inductive conditions with 180 mM KH_2_PO_4_ and 10^–4^ M dexamethasone sodium phosphate (MilliporeSigma) with or without FMX treatment. Medium was replaced every 2–3 days. After 1 week of induction, cultured cells were harvested for osteogenic marker analysis by qPCR. For alizarin red staining, cells were induced for 4 weeks, followed by staining with alizarin red to assess mineralized nodule formation.

### Statistics.

In vitro and ex vivo data are presented as mean ± standard deviation (SD) from at least 3 independent experiments. Data were analyzed using ANOVA or Kruskal-Wallis tests, with post hoc tests based on data distribution, with significance set at *P* less than 0.05. Clinical data were analyzed for outliers using Dixon’s test and are shown as mean ± SD or box plots, and Mann-Whitney *U* test was used to compare antimicrobial efficacy of FMX treatment against saline (negative) and NaOCl (positive) controls, considering *P* less than 0.05 as significant. Details are provided in the figure legends.

### Study approval.

The clinical study was conducted in accordance with the Declaration of Helsinki and approved by the Institutional Review Board of the University of Pennsylvania (no. 816238). Investigational new drug exemption for the off-label use of the FDA-approved formulation of Iron oxide nanoparticles (IONPs) (ferumoxytol) as a clinical disinfectant was obtained from the office of clinical research at the Perelman School of Medicine, University of Pennsylvania. Ethical approval and written consent/HIPAA authorization for the clinical study were obtained from the Institutional Review Board at the University of Pennsylvania (IRB 828211). The clinical study was registered on ClinicalTrials.gov (clinical trial NCT06110494).

### Data availability.

The data underlying this article are available upon request. RNA-Seq data were deposited in the Functional Genomics Data collection (ArrayExpress) with accession number E-MTAB-14616. Values underlying the graphed data are reported in the Supporting Data Values file.

## Author contributions

AB, YL, ZR, CC, AS, BK, and HK designed the research study. AB, YL, ZR, ZX, MJO, NKP, CC, and RH conducted the research study. AB, YL, ZR, ZX, RH, CC, and NKP acquired the data. AB, ZR, NKP, and CC analyzed the data. AB, YL, ZR, CC, BK, DPC, and HK participated in editing the manuscript.

## Supplementary Material

Supplemental data

Supplemental video 1

Supporting data values

## Version 1 11/26/2024

In-Press Preview

## Version 2 02/03/2025

Electronic publication
