# Supplementary material for: Ferumoxytol nanozymes effectively target chronic biofilm infections in apical periodontitis
Source: J Clin Invest. 2024 Nov 26;135(3):e183576. doi: 10.1172/JCI183576 (PMC11785919; doi:10.1172/JCI183576)
Supplement: Supplemental data [file jci-135-183576-s060.pdf]

SUPPLEMENTAL MATERIAL

Babeer et al.

Ferumoxytol nanozymes effectively target chronic biofilm infections in apical periodontitis

Table of Contents

SUPPLEMENTAL METHODS ... ***2***

Catalytic activity of FMX nanozymes ... **2**

Enterococcus faecalis **single species biofilm model (MBEC Assay®)** ... **2**

E. faecalis **biofilm treatment and analysis** ... **2**

FMX nanozymes binding and catalytic activity ... **3**

In vitro **mixed species biofilm model (MBEC Assay®)** ... **3**

In vitro **mixed-species biofilm treatment and analysis** ... **4**

FMX nanozyme binding on individual bacterial cells ... **4**

Ex vivo **mixed-species biofilm model on human natural extracted teeth** ... **4**

Ex vivo **biofilm treatment and analysis** ... **4**

Stem cells of the apical papilla apoptosis, viability and transcriptomic analysis ... **5**

Human subjects for root canal disinfection ... **5**

Inclusion criteria ... **6**

Exclusion criteria ... **6**

Clinical procedure and sample collection ... **6**

Clinical sample processing ... **7**

SUPPLEMENTAL FIGURES ... ***8***

Supplemental Figure 1. Catalytic activity of FMX Nanozymes. ... **8**

Supplemental Figure 2. Bioactivity of FMX Nanozymes against planktonic *E. faecalis*. ... **9**

Supplemental Figure 3. Bioactivity of FMX nanozymes against *E. faecalis* biofilm ... **10**

Supplemental Figure 4. Clinical study flowchart. ... **11**

Supplemental Figure 5. *In situ* catalytic activity. ... **12**

Supplemental Figure 6. Gene-set enrichment analysis for cell proliferation. ... **13**

References ... ***14***

SUPPLEMENTAL METHODS

Catalytic activity of FMX nanozymes

The peroxidase-like activity of ferumoxytol nanozymes (FMX) was tested using 3,3′,5,5′-tetramethylbenzidine (TMB) as an oxidation substrate that produces a blue color upon reacting with free radicals generated by FMX in the presence of H_2O_2. Briefly, a reaction mixture (1000 µl) containing 25, 50 or 100 µg/mL FMX nanoparticles, 3% H_2O_2 (w/w), and 100 µg/mL TMB was prepared in 0.1 M sodium acetate (NaOAc) buffer (pH 4.5). After mixing with the pipette 2 times, the colorimetric reaction was assayed spectrophotometrically (absorbance at 652 nm) using a Beckman DU800 spectrophotometer every 60 seconds for a total reaction time of 10 minutes. 3% H_2O_2 alone served as the control in the experiment. To study the effect of the presence of dental hard tissues, human enamel-dentin slabs (2 × 2 mm^2) were added to the reaction buffer immediately after mixing, and the colorimetric reaction was assayed spectrophotometrically as described above. Three independent experiments were conducted (n = 3).

Enterococcus faecalis single species biofilm model (MBEC Assay®)

Single-species *Enterococcus faecalis* (OG1RF) biofilms were formed using a commercial MBEC device that allows for rapid and reproducible biofilm antimicrobial susceptibility testing. It produces 96 highly reproducible biofilms grown on hydroxyapatite-coated (HA) pegs in a standard 96-well setup. The HA-coated pegs were vertically suspended to support biofilm growth from true bacterial adherence (rather than sedimentation on the surface). The HA coating also mimics the primary inorganic component of tooth structures.[1,2] Actively grown *E. faecalis* cells (10^9 CFU/mL) were diluted 1:10000 times in brain-heart infusion (BHI) broth supplemented with 1% glucose (w/v). Each well was inoculated with 150 µl of *E. faecalis* 10^5 CFU/mL and was incubated at 37 °C with 5% CO_2. The culture medium was changed once at 24 h. *E. faecalis* biofilms were treated and analyzed at 48 h.

E. faecalis biofilm treatment and analysis

The antimicrobial and antibiofilm activity of different concentrations of FMX/H_2O_2 against *E. faecalis* biofilms was tested in a dose- and time-dependent manner. At the end of the biofilm growth (48 h), biofilms on HA-coated pegs were dip washed in 200 µl of 0.89% NaCl to remove unbound bacteria. Biofilms were transfered into a new 96-well plate containing the treatment solution (200 µl/well), a mixture of 3% H_2O_2 (v/v) and different concentrations of FMX (0.75, 1.5, 3 and 6 mg/mL) in 0.1 M of NaOAc buffer (pH 4.5). The biofilm treatment was carried out for varying durations (1 min, 5 min, and 10 min) at 37 °C. The controls were 3% H_2O_2 alone, FMX alone (at the highest concentration, 6 mg/mL), and 0.1 M NaOAc buffer (vehicle control). All treatment solutions had a pH of 4.5. After the treatment, biofilms were dip-washed twice with 200 µL of 0.89% NaCl. Next, pegs were removed using a dental extraction forcep and each peg was transferred to glass test tubes containing 2 mL of sterile 0.89% NaCl. Biofilms were then dispersed and homogenized using a water bath sonfier (Aquasonic, 150T water bath sonifier) for 10 minutes. The pegs were removed from the glass test tube using a sterile spatula. The homogenized suspension was then transferred to a 15 mL centrifuge tube, and the glass test tube was washed 3 times with 1 mL of 0.89% NaCl. The washing solution was collected and combined into the homogenized suspension. Serial dilution from 10^{-1} to 10^{-6} was performed using sterile 0.89% NaCl, followed by plating on Trypticase Soy Agar (TSA II) blood agar plates. Experiments were done in duplicates with three independent experiments (n = 6).

In a separate experiment, antibiofilm effects of the FMX/H_2O_2 at the optimum concentration were evaluated with LIVE/DEAD assay and confocal fluorescence microscopy. After the 10-min treatment, biofilms were dip-washed twice using 200 µL of 0.89% NaCl. Pegs were then removed and mounted on 60 × 15 mm tissue culture dishes using dental wax on a heated base plate. 3.35 µM SYTO 9 (485/498 nm; Molecular Probes) and 20 µM propidium iodide (PI) (535/617 nm; Molecular Probes) were used for bacterial cell labeling in 5 mL of 0.89% NaCl. SYTO 9, a cell permeable nucleica acid dye, stains all bacteria. In contrast, PI is impermeable to cells with an intact plasma membrane and only stains killed cells with damaged membranes. When both fluorophores are present intracellularly, only PI signal is detectable due to displacement of SYTO9 by PI and quenching of SYTO9 emissions by fluorescence resonance energy transfer. After a 30-min incubation at room temperature, the staining solution was carefully aspirated, and the samples were gently washed 3 times with 0.89% NaCl. Care was taken not to dislodge the peg mounted on the wax. The samples were then submerged with 0.89% NaCl, and confocal images were acquired using

Zeiss LSM 800 upright confocal laser scanning microscope with a 20 × (numerical aperture = 1.0) water immersion objective. Three independent biological experiments were conducted and two 3D image stacks from each experiment were collected (n = 6). Image analysis was done using BiofilmQ (https://drescherlab.org/data/biofilmQ/), a fluorescence image analysis software optimized for biofilms. The raw image stacks from the SYTO 9 and PI channel were converted into BiofilmQ's TIF image format. Biofilm segmentation was done using the otsu algorithm with an optimized threshold determined by two different examiners to create binarized Z-stacks. The biofilm biovolumes from SYTO 9 (LIVE) and PI (DEAD) channels were calculated. The total biofilm biovolume was calculated using the numerical value generated from both channels, followed by calculation of the percentage of LIVE/DEAD bacteria in relation to the total biovolume.

FMX nanozymes binding and catalytic activity

To confirm FMX retention on the surface of the biofilm, quantitative assessment was performed with Inductively coupled plasma optical emission spectrometry (ICP-OES). Treated biofilms were dip washed 200 µl of 0.89% NaCl to remove unbound cells then exposed to 200 µl of 6 mg/mL of FMX in 0.1 M NaOAc (pH 4.5) for 10 minutes topically. The biofilms were dip washed twice in 200 µL 0.89% NaCl to remove unbound FMX, then pegs were removed and sonicated (water bath sonication for 10 min) to harvest the biofilm. The biomass was pelleted by centrifugation (5,500g, 10 min) and the supernatant was discarded. The pellet was then dissolved in 1 mL of concentrated HNO_3 (70%) at room temperature overnight. The next day, 9 mL of MilliQ water was added, and the sample was analyzed by ICP-OES (Spectro Genesis ICP) for iron content. Iron analytical standard was purchased from Fisher Scientific (Waltham, MA). The concentrations of iron were measured for each sample. The values obtained were used to calculate the concentration within the samples as described previously.[3] Experiments were done in duplicates from three independent experiments (n = 6).

In a separate experiment, confirmation of the binding of FMX to intact single-species biofilm was also assessed using environmental scanning electron microscopy coupled with energy dispersive spectroscopy (ESEM/EDS). 48-h biofilms were dip washed 200 µl of 0.89% NaCl to remove unbound cells then exposed to 200 µl of 6 mg/mL of FMX in 0.1 M NaOAc (pH 4.5) topically for 10 minutes. The biofilms were dip washed twice in 200 µL 0.89% NaCl to remove unbound FMX and pegs were removed as described previously. Specimens were mounted on stubs using double stick, electrically conductive copper foil tape. Samples were sputter coated with 0.1 nm thick layer of Au/Pd (gold/pallidum) using a sputter coater. Intact biofilms were examined with ESEM (Quanta 600 FEG, FEI), and the iron element were analyzed via EDS. The distribution of iron content on the biofilm surface was visualized using the elemental mapping on the same ESEM.

TMB assay was used to confirm that biofilm-bound FMX maintain their catalytic activity. Briefly, 48-h biofilms were dip washed twice in 200 µL 0.89% NaCl, then exposed to 200 µl of 6 mg/mL of FMX in 0.1 M NaOAc (pH 4.5) for 10 minutes. Next, the biofilms were dip washed twice with 0.1 M NaOAc buffer (pH 4.5) and transferred to the reaction buffer of 0.1 M NaOAc (pH 4.5) containing 100 µg/mL TMB and 3% H_2O_2. After 10 min, the colorimetric reaction was assayed spectrophotometrically with SpectraMax M2 multimode microplate reader (absorbance of 652 nm). Experiments were performed in triplicates from 4 individual experiments (n=12).

***In vitro* mixed species biofilm model (MBEC Assay®)**

Frozen stocks of *E. faecalis* (OG1RF), *Streptococcus gordonii* (DL1), and *Fusobacterium nucleatum* subspecies *nucleatum* (ATCC 28856) were streaked on TSA II Blood agar plates. The plates were placed inside an anaerobic chamber (5% CO_2, 5% H_2, and balance N_2) at 37 °C for 72 h. Mixed-species biofilms were formed on HA-coated pegs using the MBEC device. Actively growing *E. faecalis* cells (10^9 CFU/mL) and *S. gordonii* cells (10^9 CFU/mL) were diluted 1:10000; Actively growing *F. nucleatum* cells (10^9 CFU/mL) were diluted in a 1:5 dilution factor. For biofilm growth, wells were inoculated with a mixture of 10^5 CFU/mL *E. faecalis,* 10^5 CFU/mL S. *gordonii,* and 10^8 CFU/mL *F. nucleatum* in 150 µL BHI broth supplemented with 5 mg/ml yeast extract, 0.5 mg/ml L-Cysteine HCl, 5 µg/ml hemin and 0.5 µg/ml vitamin K1 (ATCC medium 1293, BHIS medium). Biofilms were grown anaerobically at 37 °C for 48 h. The culture medium was changed once at 24 h.

In vitro mixed-species biofilm treatment and analysis

At the end of the biofilm growth (48 h), biofilms were dip-washed in 200 µl of 0.89% NaCl, then treated as described above. Next, pegs were removed and transferred to pre-reduced 0.89% NaCl solution (2 mL) inside screw-cap glass tubes. Biofilms were then dispersed and homogenized by a water bath sonifier (Aquasonic, 150T) for 10 min. The sonication process was repeated for additional two times (10 min each). To avoid heating of the samples, the water inside the sonifier was replenished after each sonication cycle. The pegs were removed from the glass tube Using a sterile spatula. The homogenized suspension was processed and plated as described above. CFU analysis was done to quantify the total number of viable cells as well as species-specific viability counts. Experiments were done in duplicates with three independent experiments (n = 6).

In a separate experiment, a green fluorescent protein (GFP) tagged *E. faecalis* (OG1RF, SD 234)[4] was used together with *S. gordonii* and *F. nucleatum* to visualize killing of *E. faecalis* cells within the mixed biofilm. The pegs were then removed and were mounted on 60 × 15 mm tissue culture dishes. 20 µM PI was used to label dead cells immediately after treatment for a period of 30 minutes. The samples were washed with 0.89% NaCl and then incubated at 4 °C overnight in the presence of oxygen to recover the fluorescence signal from GFP.[5,6] The next day, samples were stained with 3.35 µM of SYTO 60 (652/678 nm; Molecular Probes) in 5 mL 0.89% NaCl for 30 minutes at room temperature The staining solution was carefully aspirated, and the samples were gently washed 3 times with 0.89% NaCl. Care was taken not to dislodge the peg mounted on the wax. The samples were then submerged with 0.89% NaCl, and confocal images were acquired as described above.

FMX nanozyme binding on individual bacterial cells

E. faecalis cells, *S. gordonii* and *F. nucleatum* were grown in BHI media anaerobically at 37 °C to late exponential phase. Subsequently, 1 mL of each bacterial cultures were centrifuged (5,500 g, 10 min) and resuspended in 6 mg/mL FMX and incubated for 10 min with rocking. After incubation, the bacteria pellets were washed twice and then dissolved in 1 mL of 70% HNO3 and finally diluted with DI water before analysis for iron quantification by inductively coupled plasma optical emission spectroscopy (ICP-OES, Spectro Genesis).

Ex vivo mixed-species biofilm model on human natural extracted teeth

We used the protocol previously reported by Hussein et al. with some modifications.[7] Single-rooted, single canal human extracted teeth were decoronated using a 0.6-mm-thick precision diamond saw (Isomet 5000; Buehler Ltd., Lake Bluff, IL) at 1000 rpm, under water cooling to create root segments with a length of 11 mm. Canals were enlarged using XP-endo 3D Shaper (Brasseler USA, Savannah, GA) at 1000 rpm followed by Endosequence files, sizes. 40 and 50 with a 4% taper (Brasseler USA, Savannah, GA). The canal space was then enlarged with Gates Glidden drill no. 1,2,3 (Tulsa Dentsply, Tulsa, OK) at 3000 rpm under water cooling to produce a canal space with a diameter of 0.9 mm. During preparation, irrigation was done using 2 mL of 3% NaOCl between files, the canals were flushed with 1 mL of 17% EDTA to remove smear layer then flushed with sterile water. The root sections were sequentially water bath sonicated in 3% NaOCl, 17% EDTA,[8] 0.89% NaCl, and BHIS media for 4 min each. The roots were extruded from the bottom of 1.5 mL Eppendorf tubes (~7 mm beyond the bottom of the tube). The apical foramen and the tooth/tube interface were coated with flowable composite PermaFlo from Ultradent (South Jordan, UT). The apparatus was placed in 15 mL tubes, filled with 1 mL of BHIS media, and autoclaved at 120 °C for 15 min. The samples were kept inside an anaerobic chamber at 37 °C for 24 h for reduction and to confirm sterility. To form mixed-species biofilms, teeth were inoculated with 1 mL of 10^5 CFU/mL of *E. faecalis,* 10^5 CFU/mL of S. *gordonii,* and 10^8 CFU/mL of *F. nucleatum* in BHIS media. Biofilms were grown anaerobically at 37 °C for 3,7, and 21 days. Growth media was changed at 24 h, then every 48 h until the end of the growth period (7 days).

Ex vivo biofilm treatment and analysis

To evaluate the effect of FMX/H_2O_2, after the growth period, the media was aspirated from the apparatus, and the resin interface was wiped with a sterile cotton applicator. Biofilms were treated for 10 min with 2 mL 0.89% NaCl, 3% NaOCl, or FMX/H_2O_2. The treatment was done by exposing the biofilm to 1 mL of the treatment solution applied at a rate of 1 mL/min and kept inside the canal for a total exposure time of 5 minutes. The treatment solution was removed, and another 1mL was added at the same rate for a second 5 min. The treatments were applied using a sterile 3 mL Luer-Lok syringes from BD Biosciences (Franklin Lakes, NJ) and 31-gauge Navitip needles from Ultradent

(South Jordan, UT). The needle was placed at the full length of the canal during the application of treatment. Biofilms were washed twice with 2 mL 0.89% NaCl after treatment, and 100 µl of BHIS media was introduced inside the canal using gel loading tips and agitated using a 50/0.04 endodontic treatment file for 1 min. The media was then collected using a gel loading tip, serially diluted in BHIS media, plated on TSA II blood agar plates, and incubated at 37 °C anaerobically for 5 days.

Stem cells of the apical papilla apoptosis, viability and transcriptomic analysis

To detect cell apoptosis, human SCAPs (0.1×10^6 cells per well) were seeded on 12-well culture plates. After cell attachment, FMX were loaded for 30 min, washed with PBS, and then continued culture for another 12 h. For low dosage long-term culture, 0.1×10^6 SCAPs were seeded on 12-well culture plates, followed by FMX nanoparticle treatment for 24 h. Apoptotic SCAPs were immunostained by Annexin V Apoptotic Detection Kit (BD Bioscience) and analyzed by FACSCalibur flow cytometer with CellQuest software. In addition, SCAPs with FMX nanoparticles treatment were stained with Live/Dead two-color staining kit (Invitrogen) and imaged to determine the viability of cells. Green-fluorescent calcein-AM was used to indicate intracellular esterase activity and red-fluorescent ethidium homodimer-1 was performed to indicate loss of plasma membrane integrity. For Ki67 staining, 1×10^4 SCAPs were seeded on 2-well chamber slides (Nunc) and cultured for 60%-70% confluence with or without FMX treatment. Then the cultures were fixed and incubated with Ki67 antibody (1:200, Cell Signaling Technology) for overnight at 4°C, followed by 1-hour secondary antibody staining. The number of Ki67-positive cells was indicated as a percentage of the total cell number.For Transcriptomics analysis, total RNA was isolated by TRIZOL reagent and miRNeasy Mini Kit (Qiagen) from the cultured cells with or with FMX treatment according to the manufacturer's instructions. RNA samples were then shipped to Novogene Corp. for library preparation, fragmentation, cDNA synthesis, second-strand synthesis, PCR enrichment, and sequencing by Illumina sequencers. Reads obtained from RNA-seq were aligned to the human reference genome. The differential expression between conditions was statistically assessed, and genes with FC > 1 and FC < 1 and P value < 0.01 were identified as differentially expressed. GSEA analysis was performed by using GSEA software with differentially expressed genes. For real-time qPCR analysis, SuperScript III Reverse Transcriptase kit (Invitrogen) was used to prepare the cDNA. qPCR assays were then performed using SYBR Green Supermix (Bio-Rad) and gene specific primer pairs, which were synthesized and purified by Integrated DNA Technologies. The RNA expression was finally normalized to GAPDH. A CFX96 Real-Time PCR System (Bio-Rad) was used for qPCR analysis.

Human subjects for root canal disinfection

IND exemption for the off-label use of the FDA-approved formulation of IONPs (ferumoxytol) in a single treatment as a clinical disinfectant was obtained from the office of clinical research at Perelman School of Medicine, University of Pennsylvania. Ethical approval of the study and the combined written consent/HIPAA authorization forms were obtained from Institutional Review Board (IRB) at the University of Pennsylvania (IRB 828211), Clinical trial number NCT06110494. The sample size estimate was calculated in Pass Software 2021, using a test that compares the ratio of two means from independent samples using data that has been log-normalized. An alpha of 0.05 and power of 80% was assumed, with means and standard deviations pulled from previous studies that employed similar methodology.[9–12] This produced a required sample size of 16 for each group. The clinical study started with a pilot phase for initial evaluation of sampling methods and initial clinical assessment of FMX/H_2O_2 approach. Forty-four (44) patients presenting to the Department of Endodontics for evaluation and treatment were assessed for eligibility and were asked if they want to participate in the study. The informed consent was obtained by the principal investigator accordingly. In the following phase, patients went through randomization and equal proportion allocation by the process of drawing lots done by an independent investigator (Z.R). The same investigator (Z.R.) prepared the syringes for the allocated treatment and placed them in a closed envelope. (Z.R.) maintained the drawing lot box and treatment allocations in a locked box. The principal investigator (A.B.) was responsible for obtaining the samples during the clinical procedure.

The clinical procedure was done by residents of the Department of Endodontics at the University of Pennsylvania School of Dental Medicine, who were calibrated to conduct the procedure following the study protocol. The principal investigator and the clinicians were blinded to the treatment allocation until the closed envelope was opened during the treatment and sample collection. At that point, it was impossible to blind the clinician and the investigator due to the clear difference in color between the treatments. In contrast, the patients remained blinded throughout the study period.

Inclusion criteria

1. Patients are willing to participate in the study.
2. Patients are 18 years or above.
3. Non-contributory medical history (Patient can be seen for regular dental appointments at PDM; ASA classes I and II).
4. Tooth requiring root canal treatment with radiographic presence of periapical radiolucency and responding to thermal sensitivity testing negatively (difluordichlormethane at 50 °C) (Endo-Ice, Coltène/Whaledent Inc., Cuyahoga Falls, Ohio) and negatively to EPT testing.
5. Tooth with adequate remaining tooth structure for proper isolation with rubber dam.
6. No history of previous endodontic treatment on the tooth.
7. Teeth with single canal and single and roots with single canals in multirooted teeth.

Exclusion criteria

1. Self-reported pregnancy.
2. Patients requiring antibiotic premedication prior to dental treatment.
3. Patients with multiple drug allergies.
4. Patients with known hypersensitivity to ferumoxytol or any iron products.
5. Patients who are scheduled for MRI for the head region within three months after ferumoxytol application.
6. Periodontal changes (pockets 3 mm, mobility Grade I or gingival edema).
7. Radiographic presence of resorptive processes.
8. Cracked and fractured teeth.
9. If one of the inclusion criteria is not met.

Clinical procedure and sample collection

The patients were anesthetized, and the tooth was isolated with rubber dam and opal dam was placed around the tooth. The tooth, the clamp, and the rubber dam were cleansed using 30% hydrogen peroxide and disinfected with 3% NaOCl. After access preparation with sterile burs and sterile saline irrigation, thermoplastic gutta percha was placed to temporarily block the orifice. The field, including the pulp chamber, was cleaned and disinfected as above. NaOCl was neutralized with 10% sodium thiosulfate. After removal of the gutta percha from the root

canal orifices and working length determination, liquid dental transport media (LDT) was in the canal with a sterile 31g needle, and a #15 Hedstrom file was used in a filing motion to scrape the canal walls and agitate the canal content.[9] (S1) sample was taken by placing a sterile paper point in the canal, allowed it to saturate for 30 sec, and then transferred to a tube containing LDT. This step was repeated once more with a second paper point. The treatment solution (saline, NaOCl, or FMX/H_2O_2) 2 mL was introduced into the canal. Canals were instrumented with 15/0.04, 20/0.04, and 25/0.04 using 2 mL of treatment solution after each file with a total of 8 mL and a total contact time of 10 min. Canal contents were deactivated with sodium thiosulphate for NaOCl, and saline wash was used for FMX/H_2O_2 and saline treatments. A wash step with 1 mL saline was done to wash the deactivating solution, and paper points were used to dry the canals. A second bacterial sample was taken (S2) by placing LDT inside the canal, agitating it with 25/0.02 Hedstrom hand file, followed by absorbing the content with 2 paper points placed in the canal for 30 sec each. The paper points were placed inside a tube containing LDT.

Clinical sample processing

Samples were immediately taken to the lab. The samples were vortexed and serially diluted from 10^{-1} to 10^{-4} using glass tubes containing 1mL of LDT. Brucella blood agar (item no. AS-614, Anaerobe Systems, Morgan Hill, CA) was inoculated with 100 μL of undiluted sample, as well as each of the three dilutions. The plates were incubated at 37°C in an anaerobic chamber for 7 days. After incubation, the number of colony-forming units (CFU) was counted by an independent investigator (Z.X) to ensure blinding other investigators (A.B.) and (Z.R) to the results until the study was concluded.

SUPPLEMENTAL FIGURES

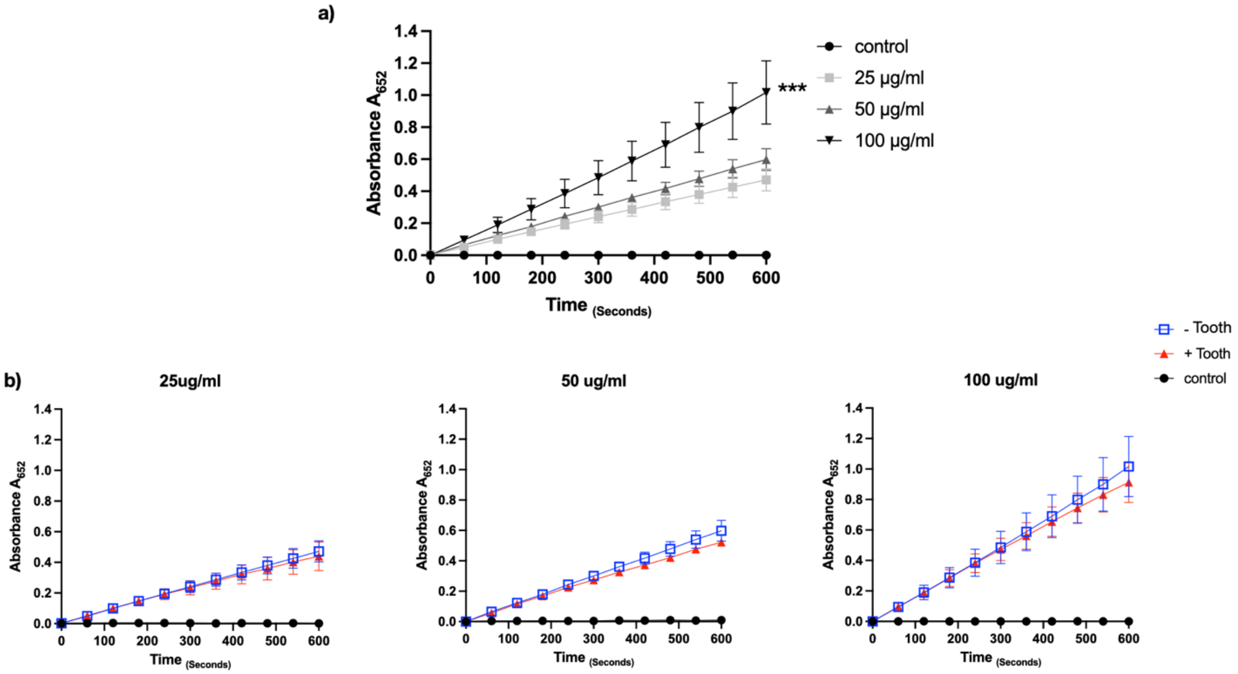

Supplemental Figure 1. Catalytic activity of FMX Nanozymes. a) catalytic activity of FMX nanozymes at different concentrations showing a dose-dependent effect over time. Data are presented as mean ± standard deviation from three independent experiments (n= 3). b) catalytic activity of FMX at different concentrations in the presence of dental hard tissues. Data are mean ± standard deviation from three independent experiments (n= 3). The quantitative data were subjected to one-way ANOVA and Tukey's test for multiple comparisons (***$P <$ 0.001).

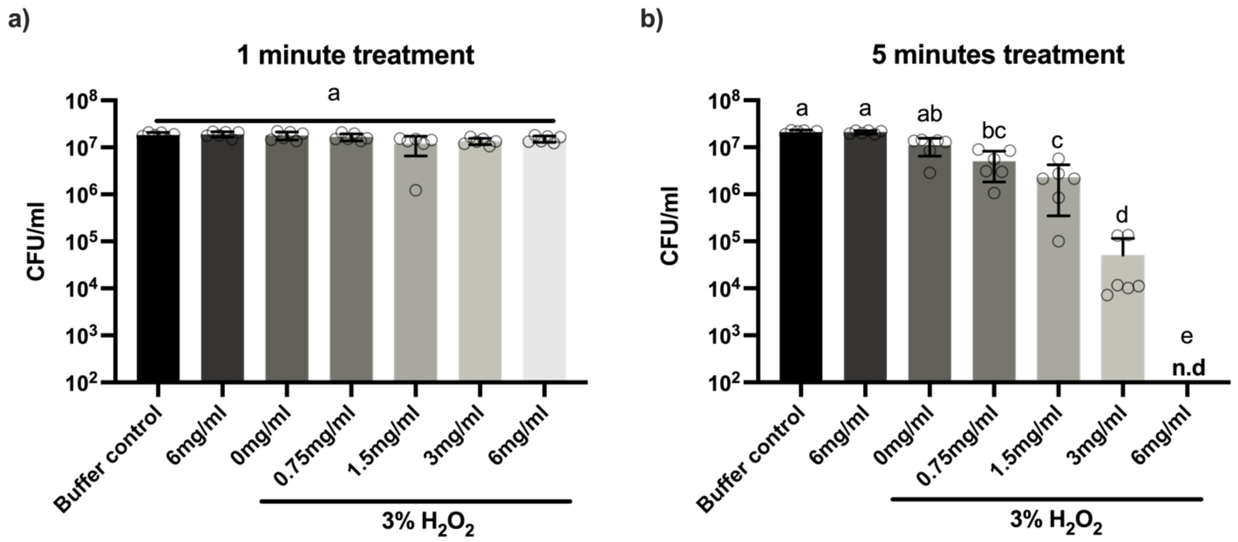

Supplemental Figure 2. Bioactivity of FMX Nanozymes against planktonic *E. faecalis*. a) Dose-dependent effect of FMX/H_2O_2 after 1-min treatment. b) Dose-dependent effect of FMX/H_2O_2 after 5-min treatment. Data are presented as the mean ± standard deviation from three independent experiments (n = 6) for each condition. The quantitative data were log 10 transformed and subjected to one-way ANOVA and Tukey's test for multiple comparisons. Significant level Significant level was set at $P < 0.05$. Differences are represented by different letters where groups assigned the same letter are not statistically different.

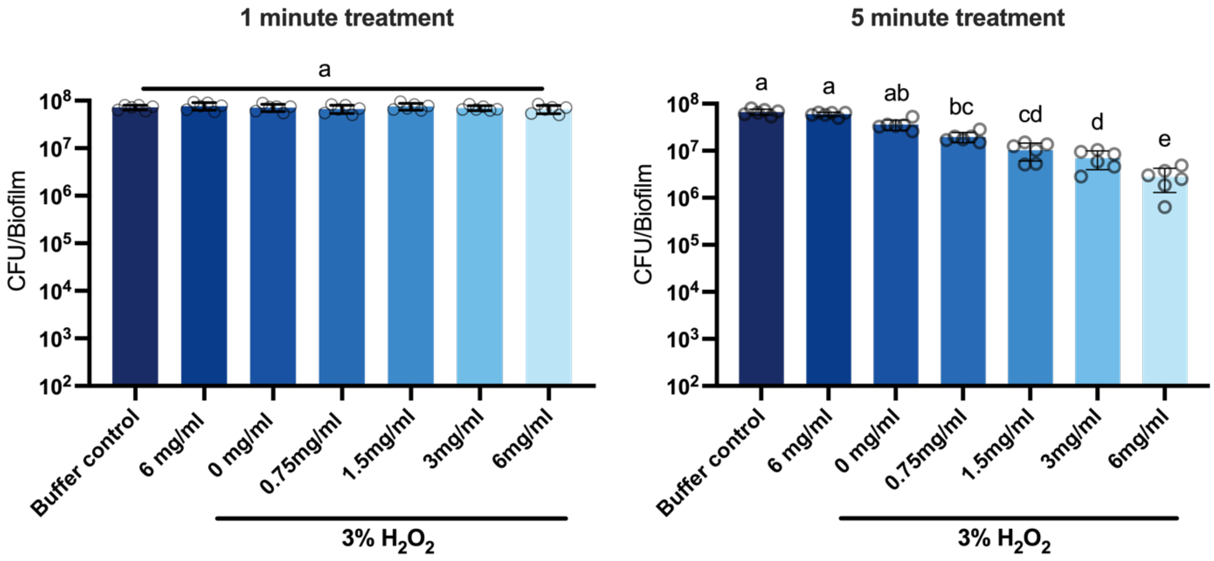

Supplemental Figure 3. Bioactivity of FMX nanozymes against *E. faecalis* biofilm. a) Dose-dependent effect of FMX/H_2O_2 after 1-min treatment. b) Dose-dependent effect of FMX/H_2O_2 after 5-min treatment. Data are presented as the mean ± standard deviation from three independent experiments (n = 6) for each condition. The quantitative data were log 10 transformed and subjected to one-way ANOVA and Tukey's test for multiple comparisons. Significant level was set at $P < 0.05$. Differences are represented by different letters where groups assigned the same letter are not statistically different.

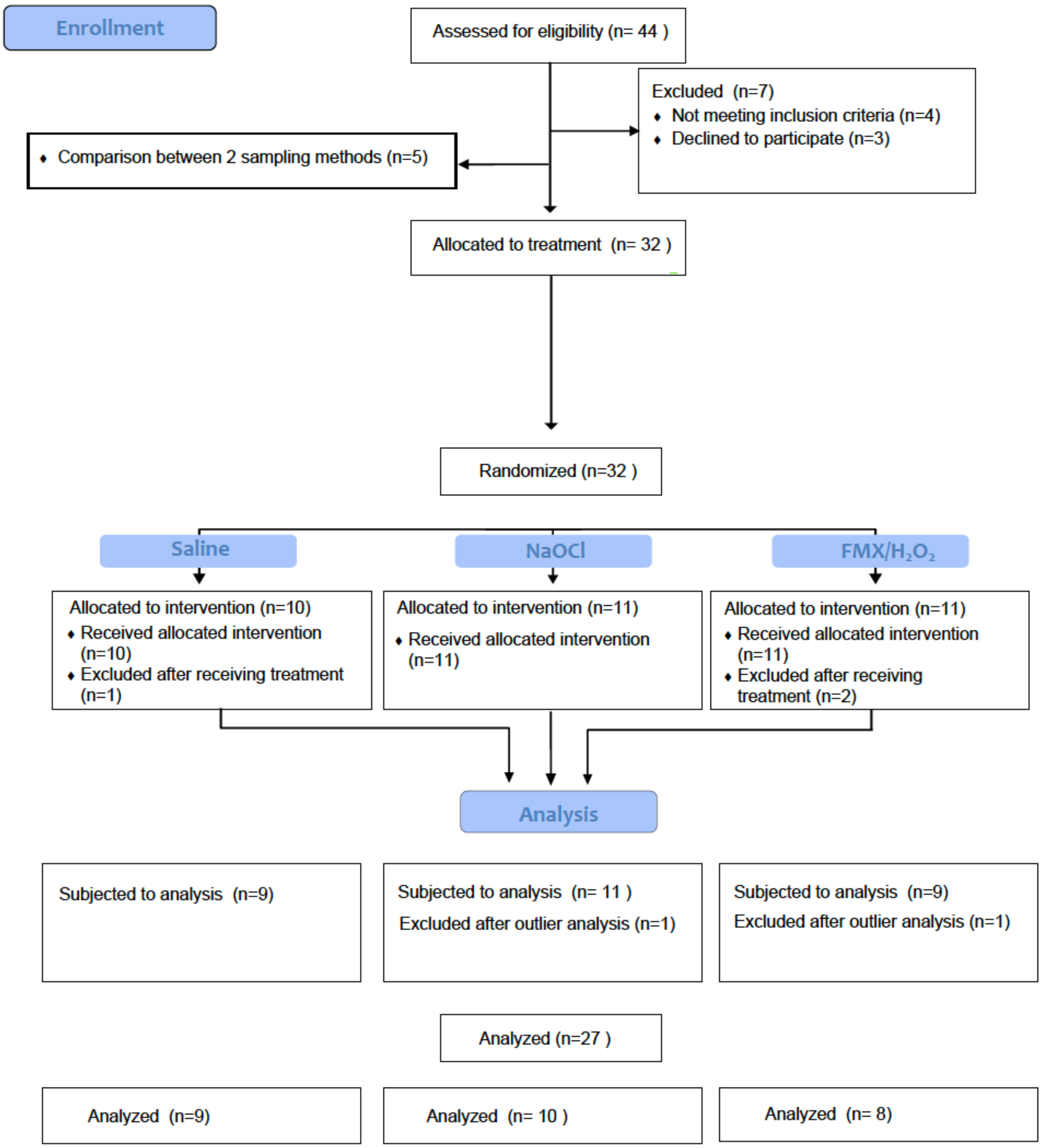

Supplemental Figure 4. Clinical study flowchart. Forty-four (44) subjects were assessed for eligibility. Seven (7) subjects were initially excluded during the eligibility assessment (not meeting inclusion criteria (n=4); declined to participate (n=3)). An initial phase of the clinical study included a comparison between paper point and endodontic file sampling to determine the method with a higher yield of colony forming units (n=5) (data not shown). Thirty-two (32) subjects who consented to participate in the study were randomly allocated to one of the 3 treatment groups through the process of drawing lots from a box that was maintained in a locked cabinet. For the saline treatment (negative control), Ten (10) subjects were allocated to and received the intervention (n=10). One sample was excluded after receiving the treatment because of processing error. The total number of samples that were included in the analysis of the saline group was (n=9). For NaOCl treatment (positive control), eleven (11) patients were allocated to and received the intervention. One sample was excluded after receiving the treatment based on Dixon's outlier test. The total number of samples that were included in the analysis of the NaOCl group was (n=10). As for the FMX/H_2O_2, eleven (11) subjects were allocated to and received the intervention. One sample was excluded because it did not meet the meet the inclusion criteria #7 (n=1), and two samples were excluded due to processing error (n=1) and not meeting Dixon's outlier test (n=1). The total number of samples included in the analysis of the FMX/H_2O_2 group was (n=8).

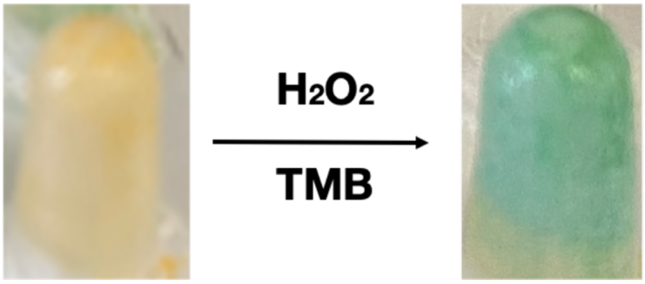

Supplemental Figure 5. *In situ* catalytic activity. Photographic images of ferumoxytol treated biofilm before and after exposure to H_2O_2 in the presence of TMB. The blue color indicates free-radical generation via H_2O_2 catalysis *in situ*.

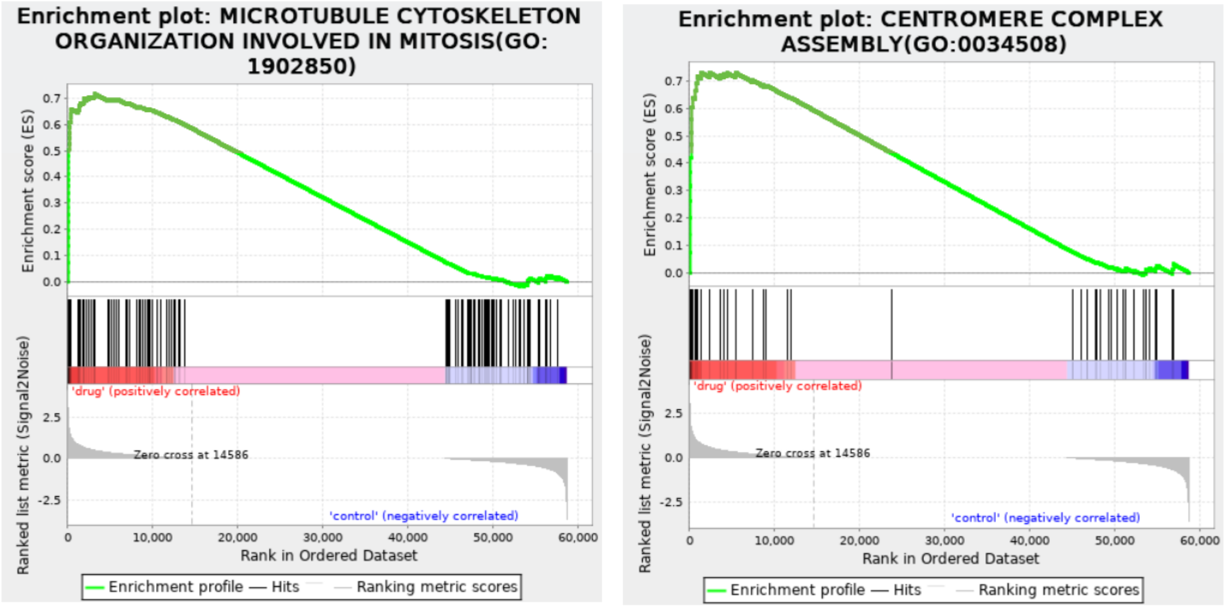

Supplemental Figure 6. Gene-set enrichment analysis for cell proliferation. Transcriptomics analysis of FMX-treated SCAPs were interrogated by gene-set enrichment analysis (GSEA) to identify enriched pathways.

References

1 Arias-Moliz MT, Ferrer-Luque CM, Espigares-García M, Baca P. *Enterococcus faecalis* biofilms eradication by root canal irrigants. *Journal of Endodontics* 2009; **35**: 711–4.

2 Arias-Moliz MT, Ferrer-Luque CM, González-Rodríguez MP, Valderrama MJ, Baca P. Eradication of *Enterococcus faecalis* biofilms by cetrimide and chlorhexidine. *Journal of Endodontics* 2010; **36**: 87–90.

3 Naha PC, Al Zaki A, Hecht E, *et al.* Dextran coated bismuth–iron oxide nanohybrid contrast agents for computed tomography and magnetic resonance imaging. *Journal of Materials Chemistry B* 2014; **2**: 8239–48.

4 DebRoy S, van der Hoeven R, Singh KV, *et al.* Development of a genomic site for gene integration and expression in *Enterococcus faecalis*. *Journal of Microbiological Methods* 2012; **90**: 1–8.

5 Cubitt AB, Heim R, Adams SR, Boyd AE, Gross LA, Tsien RY. Understanding, improving and using green fluorescent proteins. *Trends in Biochemical Sciences* 1995; **20**: 448–55.

6 Scott KP, Mercer DK, Glover LA, Flint HJ. The green fluorescent protein as a visible marker for lactic acid bacteria in complex ecosystems. *FEMS Microbiology Ecology* 1998; **26**: 219–30.

7 Hussein H, Kishen A. Antibiofilm and immune response of engineered bioactive nanoparticles for endodontic disinfection. *Journal of Clinical Medicine* 2020; **9**: 730.

8 Zapata RO, Bramante CM, de Moraes IG, *et al.* Confocal laser scanning microscopy is appropriate to detect viability of *Enterococcus faecalis* in infected dentin. *Journal of Endodontics* 2008; **34**: 1198–201.

9 Dalton BC, Ørstavik D, Phillips C, Pettiette M, Trope M. Bacterial reduction with nickel-titanium rotary instrumentation. *Journal of Endodontics* 1998; **24**: 763–7.

10 Shuping G, Orstavik D, Sigurdsson A, Trope M. Reduction of intracanal bacteria using nickel-titanium rotary instrumentation and various medications. *Journal of Endodontics* 2000; **26**: 751–5.

11 Siqueirajr J, Guimaraespinto T, Rocas I. Effects of chemomechanical preparation with 2.5% sodium hypochlorite and intracanal medication with calcium hydroxide on cultivable bacteria in infected root canals. *Journal of Endodontics* 2007; **33**: 800–5.

12 Aveiro E, Chiarelli-Neto VM, de-Jesus-Soares A, *et al.* Efficacy of reciprocating and ultrasonic activation of 6% sodium hypochlorite in the reduction of microbial content and virulence factors in teeth with primary endodontic infection. *International Endodontic Journal* 2020; **53**: 604–18.
